# Supplementary material for: Physiological and Antioxidant Response to Different Water Deficit Regimes of Flag Leaves and Ears of Wheat Grown under Combined Elevated CO2 and High Temperature
Source: Plants (Basel). 2022 Sep 13;11(18):2384. doi: 10.3390/plants11182384 (PMC9504337; doi:10.3390/plants11182384)
Supplement: Supplementary file 1 [file plants-11-02384-s001.zip › Supplementary Figures_plants-1881609.pdf]

Supplementary Figures by Bendou et al (2022)_plants-1881609

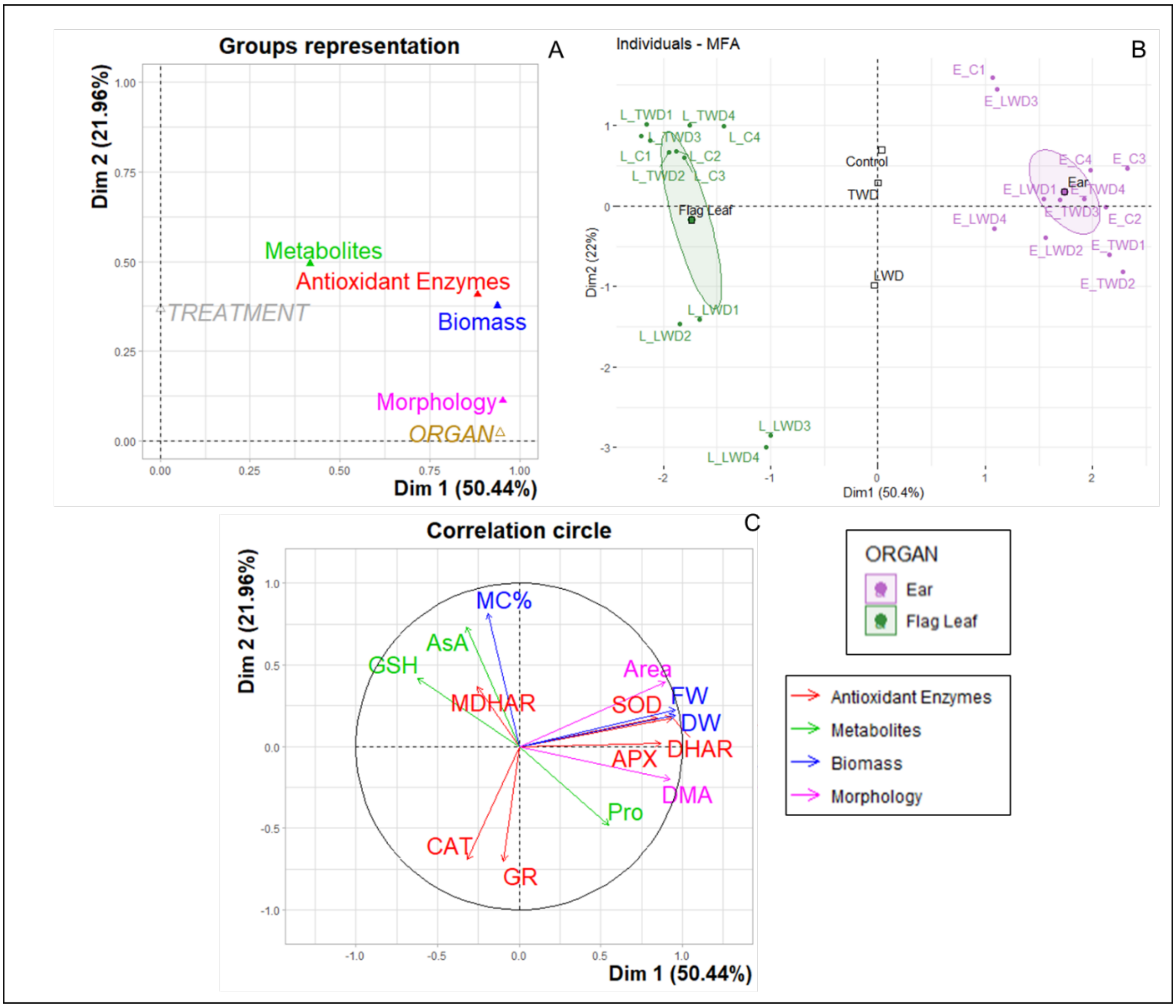

Figure S1. Multiple factorial analysis for organ and different water regimes in wheat grown under elevated CO_2 and high temperature. *Dim*, Dimension; DW, dry weight; FW, fresh weight; MC%, moisture content percentage; Pro, proline content; ASA, total content of ascorbate (AsA plus DHA); GSH, total content of glutathione (GSH plus GSSG); SOD, superoxide dismutase; CAT, catalase; APX, ascorbate peroxidase; DHAR, dehydroascorbate reductase; MDHAR, monodehydroascorbate reductase; GR, glutathione reductase. Two factors were defined: *ORGAN*, which included flag leaf and ear data, and *TREATMENT*, which included control, long water deficit (LWD) and terminal water deficit (TWD). The green biomass and biochemical traits were divided into four groups: *Antioxidant Enzymes* (CAT, SOD, APX, DHAR, MDHAR and GR), *Biomass* (FW, DW and MC%), *Metabolites* (Pro, AsA and GSH), *Morphology* (Area and DMA). Panels A contains the group representation plot showing the correlation between the trait groups of the continuous variables and the two factors with the first and second dimensions of the MFA axes. Triangles represent the relative position of each group with the axes. Panel B contains the correlation circle plot showing the correlation of variables with the MFA axes. Panel C contains the individuals-MFA plot showing the position of the individuals in the MFA by organ and treatment. Dots indicate individuals. Ellipses around each organ were added.

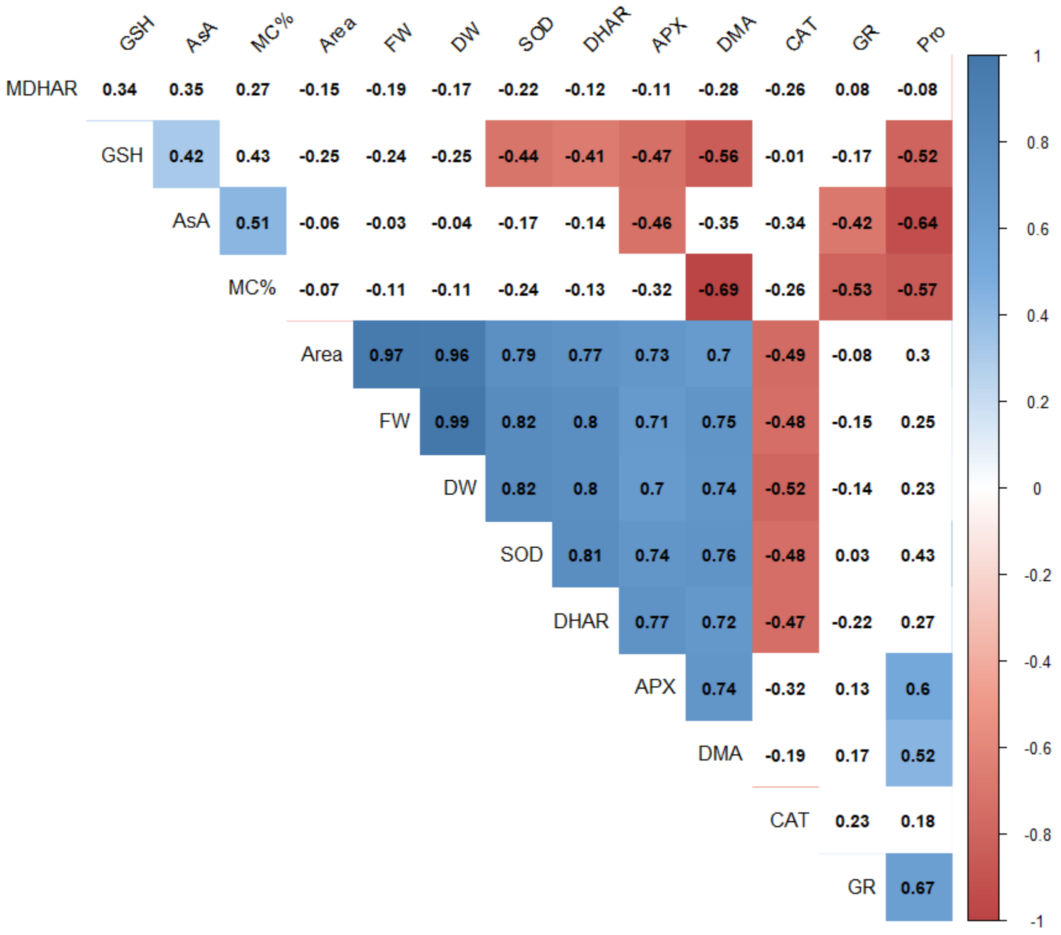

Figure S2. Correlogram for the biomass-, morphology-, metabolite- and antioxidant enzyme-related variables in the flag leaf and the ear of potted wheat plants subjected to long and terminal water deficit (LWD and TWD) regimes under elevated CO_2 and high temperature. Data (r_s) was generated from Spearman correlation analysis. Statically significant correlations (p-value ≤ 0.05) were coloured. Continuous variable abbreviations are defined in the legend of Figure S1.

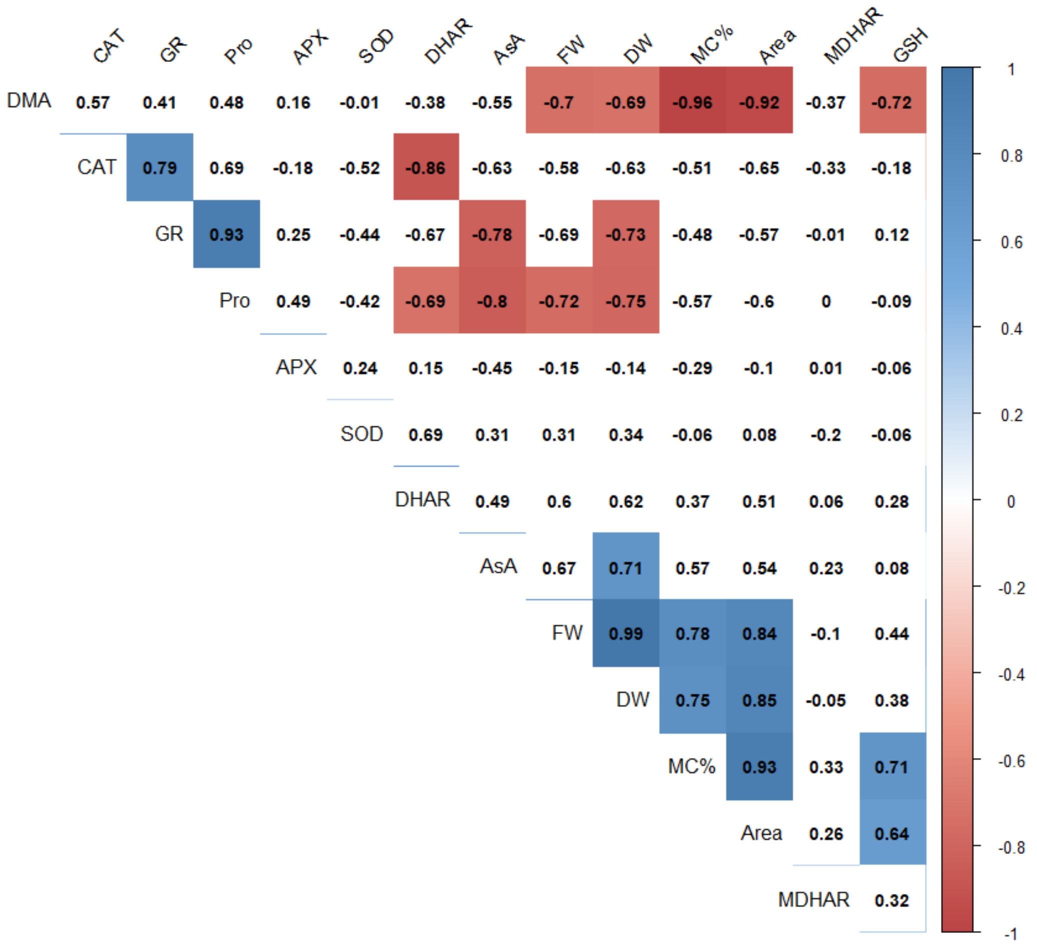

Figure S3. Correlogram for the biomass-, morphology-, metabolite- and antioxidant enzyme-related variables in the flag leaf of potted wheat plants subjected to long and terminal water deficit (LWD and TWD) regimes under elevated CO_2 and high temperature. Data (r_s) was generated from Spearman correlation analysis. Statically significant correlations (p-value ≤ 0.05) were coloured. Continuous variable abbreviations are defined in the legend of Figure S1.

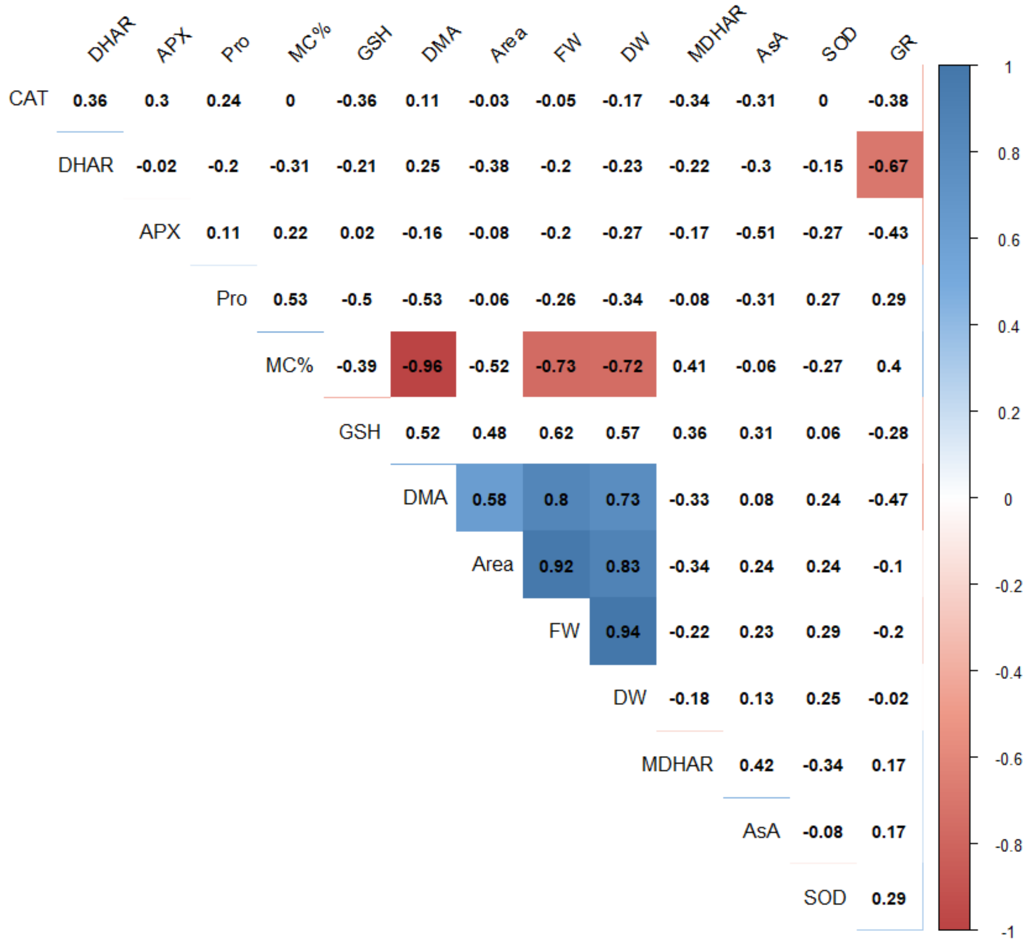

Figure S4. Correlogram for the biomass-, morphology-, metabolite- and antioxidant enzyme-related variables in the ear of potted wheat plants subjected to long and terminal water deficit (LWD and TWD) regimes under elevated CO_2 and high temperature. Data (r_s) was generated from Spearman correlation analysis. Statically significant correlations (p-value ≤ 0.05) were coloured. Continuous variable abbreviations are defined in the legend of Figure S1.
